# Multichromatic near-infrared imaging to assess interstitial lymphatic and venous uptake *in vivo*

**DOI:** 10.1117/1.JBO.26.12.126001

**Published:** 2021-12-08

**Authors:** Fabrice C. Bernard, Jarred Kaiser, Sarvgna K. Raval, Zhanna V. Nepiyushchikh, Thanh N. Doan, Nick J. Willett, J. Brandon Dixon

**Affiliations:** a Georgia Institute of Technology and Emory University, Wallace H. Coulter Department of Biomedical Engineering, Atlanta, Georgia, United States; b Emory University, Department of Orthopaedics, Atlanta, Georgia, United States; c Georgia Institute of Technology, George W. Woodruff School of Mechanical Engineering, Atlanta, Georgia, United States; d Atlanta Veteran’s Affairs Medical Center, Department of Orthopaedics, Atlanta, Georgia, United States; e Georgia Institute of Technology, Parker H. Petit Institute for Bioengineering and Bioscience, Atlanta, Georgia, United States

**Keywords:** tissue optics, NIR imaging, venous, lymphatic, clearance

## Abstract

**Significance:** Changes in interstitial fluid clearance are implicated in many diseases. Using near-infrared (NIR) imaging with properly sized tracers could enhance our understanding of how venous and lymphatic drainage are involved in disease progression or enhance drug delivery strategies.

**Aim:** We investigated multichromatic NIR imaging with multiple tracers to assess *in vivo* microvascular clearance kinetics and pathways in different tissue spaces.

**Approach:** We used a chemically inert IR Dye 800CW (D800) to target venous capillaries and a purified conjugate of IR dye 680RD with 40 kDa PEG (P40D680) to target lymphatic capillaries *in vivo*. Optical imaging settings were validated and tuned *in vitro* using tissue phantoms. We investigated multichromatic NIR imaging’s utility in two *in vivo* tissue beds: the mouse tail and rat knee joint. We then tested the ability of the approach to detect interstitial fluid perturbations due to exercise.

**Results:** In an *in vitro* simulated tissue environment, free dye and PEG mixture allowed for simultaneous detection without interference. In the mouse tail, co-injected NIR tracers cleared from the interstitial space via distinct routes, suggestive of lymphatic and venous uptake mechanisms. In the rat knee, we determined that exercise after injection transiently increased lymphatic drainage as measured by lower normalized intensity immediately after exercise, whereas exercise pre-injection exhibited a transient delay in clearance from the joint.

**Conclusions:** NIR imaging enables simultaneous imaging of lymphatic and venous-mediated fluid clearance with great sensitivity and can be used to measure temporal changes in clearance rates and pathways.

## 1 Introduction

The circulatory system maintains tissue homeostasis through the continuous delivery of nutrients and oxygen to the tissue space and the removal of proteins and waste products. Crucial to this process is the removal of interstitial fluid, proteins, and lipids by the lymphatic vasculature; this fluid is returned to circulation through absorption at lymph nodes and delivery to the venous system through lymphatic ducts. Recent developments in optical imaging have provided new capabilities to quantify lymphatic function *in vivo*.

In general, there are two routes of fluid clearance from tissues: (1) venous uptake and (2) lymphatic uptake. Venous return in tissue beds is passive, size-dependent, and varies based on capillary physiology.^1^^,^^2^ In contrast, lymphatic capillaries originate from the tissues and have flap-like openings that nondiscriminately allow molecules of all sizes to enter. The extrinsic motion of the surrounding tissue combined with the intrinsic contractility of downstream lymphatics, create transient pressure gradients that allow fluid and macromolecules to enter the vessel and be transported downstream. Impaired interstitial fluid clearance has been implicated in various diseases, including lymphedema,^3^ cancer,^4^ and arthritis.^5^ Techniques to measure clearance kinetics from interstitial spaces are critical to evaluating disease state and different tissues’ ability to drain molecules from the interstitial spaces. These measurements have been assessed classically via radiolabeled agents, which require serial collection of fluids, carry potential toxicity, and require additional safety measures.^6^^–^^8^ However, the advent of near-infrared (NIR) fluorescent imaging allows for less invasive, cost-effective, high resolution, clinical, and preclinical imaging in a variety of applications.^9^^–^^11^

NIR-based technologies have advanced considerably in the last decade—both in terms of imaging components as well as tracers and fluorophore-based probes—which have allowed for significant new *in vivo* capabilities. The NIR imaging window includes the visible and infrared light spectrum from 650 to 1300 nm, which due to decreased scattering and absorption coefficients, penetrates tissues deeper than higher energy light.^12^^–^^14^ Contrast agents such as indocyanine green (ICG), polyethylene glycol (PEG) conjugated with NIR dyes, or NIR quantum dots have been used to visualize lymphatics and blood vessels *in vivo*.^15^^–^^19^ Preclinical NIR imaging has also previously enabled the measurement of tracers’ differential uptake as a function of size from different tissue beds.^17^^,^^20^ Multichromatic NIR imaging (e.g., imaging with multiple NIR fluorescent probes) empowers mapping of drainage zone of lymph nodes in rodents.^19^^,^^21^^,^^22^ However, this has not yet been widely extended to differentiate between venous uptake and lymphatic uptake simultaneously from the same tissue bed. Recently, in humans, multispectral optoacoustic tomography has been used to visualize lymphatics and blood vessels in three-dimensional.^23^ While this technique can be used for imaging the architecture of the two vessel networks, a feature that would be particularly useful for guided lymphatic microsurgery, it does not provide functional assessment of lymphatic contractility.

We have previously shown the size-dependent uptake of 2- and 40-kDa NIR-PEG into the venous and lymphatic circulation, respectively, after intra-articular injection into the rat knee joint.^24^ Further, we demonstrated that endothelin-1 (ET-1), a vasoactive compound, transiently reduced the outflow of both PEG tracers from the joint in a dose-dependent manner. Due to these experiments’ monochromatic nature, we were unable to assess lymphatic and venous drainage simultaneously. The inability to differentiate clearance mechanisms and function between the venous and lymphatic systems is a critical technological gap that has broad implications for many different tissues and disease states. Coupling *in vivo* delivery with multichromatic NIR imaging could allow for the advancement of the understanding of how the venous and lymphatic drainage may change in the context of diseases or physical interventions. The objective of this study was to develop a technological approach that couples NIR imaging with the size-dependent clearance of tracers *in vivo*. We hypothesized that a multichromatic imaging approach for differentially imaging the lymphatic and venous systems would show the technique’s utility in both the mouse tail, where the superficial vessels can be visualized, and in the rat knee joint, where drainage occurs slowly within deeper tissue structures. In addition, we perturbed the joint microenvironment by exercising the rats on a treadmill and detected changes to venous and lymphatic clearances within the knee joint.

## 2 Materials and Methods

### 2.1 Tracers for *In Vitro and In Vivo* Studies

IR dye 800CW carboxylate (D800) (LI-COR Biosciences) was purchased as a lyophilized powder. Twenty nanomoles were resuspended in 100  μl of sterile saline to make a 20-mM stock solution. For tissue phantom studies and knee injections, the stock solution was diluted to 0.4 mM in sterile saline.

40-kDa methoxy PEG amine (JenKem Technology) was purchased as lyophilized powder. To conjugate 40-kDa PEG amine to IR Dye 680RD, 16 mg of PEG amine was reacted with 30  μl of 10  mg/ml IR Dye 680RD NHS ester [diluted in dimethyl sulfoxide (DMSO)] in a total of 1 ml of Dulbecco’s phosphate-buffered saline overnight. Excess IR dye, salts, and DMSO were removed via centrifugal filtration using 10-kDa molecular weight cutoff centrifugal filters (Amicon Ultra) and three consecutive washes were conducted with deionized water. The purified tracers were aliquoted into 10 equal volumes of 100  μl, lyophilized, and kept at −20°C. For tail injections, lyophilized aliquots were resuspended in 100  μl of sterile saline, and 2.5  μl of P40D680 was mixed with D800 for intradermal injection. For tissue phantom studies and knee clearance studies, lyophilized aliquots were resuspended in 100  μl.

### 2.2 Optical Properties of Tracers

To quantify each tracer’s absorbance in the visible and NIR range, 0.4 mM of D800 and 1  mg/mL of P40D680 were scanned in a spectrophotometer (Ultrospec 2100, Biochrom). The emission and excitation spectra of D800 and P40D680 were assessed using a microplate reader with filter-based emission and detection capabilities (Synergy H4, BioTek). For P40D680 and D800, fixed emission filters of 720 and 840 nm were used for excitation scan from 400 to 700 nm and 400 to 820 nm, respectively. Fixed excitation wavelengths of 660 and 760 nm were used to conduct an emission scan from 680 to 900 nm for P40D680 and from 780 to 900 nm for D800.

### 2.3 NIR Imaging Setup

Multichromatic NIR imaging was carried out using a customized NIR setup.^17^^,^^25^ Briefly, the system consisted of a cooled EMCCD camera (Evolve eXcelon, Photometrics) attached to a stereomicroscope with adjustable zoom (MVX10, Olympus), a shutter-controlled xenon arc light source (Lambda LS, Sutter Instrument Company), and a manual-operated filter wheel equipped with two filters: (1) a standard Cy5.5 filter cube (635 to 675 nm excitation, 696 to 736 nm emission) and (2) an ICG-B filter cube (748 to 789 nm excitation, 814 to 851 nm emission) (Chroma Technology). The electronic shutter was left open during continuous imaging, and images were acquired using MicroManager software.^26^

### 2.4 Dye and Tracer Characterization and Tissue Phantom Studies

Polydimethylsiloxane (PDMS) tissue phantoms, measuring 2 and 4 mm in thickness, were created as previously described.^15^ Briefly, tissue phantoms were composed by weight of 88.10% silicone elastomer base (Sylgard 184, Dow Corning) mixed with 8.81% curing agent (Sylgard 184, Dow Corning), 1.76% aluminum oxide (Sigma Aldrich), and 1.32% cosmetic powder (Max Factor Crème Puff Deep Beige 42). PDMS phantoms were poured into plastic mold and cured in the oven at 60°C overnight.

Stock P40D680 and D800 were diluted serially in twofold dilutions with PBS. Tissue phantoms were used to mimic tissue depth and its effect on tracer fluorescence intensity. The 2-mm tissue phantom was used to mimic the superficial collecting vessels in mouse tail, and 4 mm tissue phantom was chosen to mimic the depth of rat knee. All images were taken with an exposure time of 50 and 5 ms, respectively, for D800 and P40D680.

### 2.5 Tail Injections to Visualize and Quantify Routes of Tracer Clearance

**Fig. 1:**
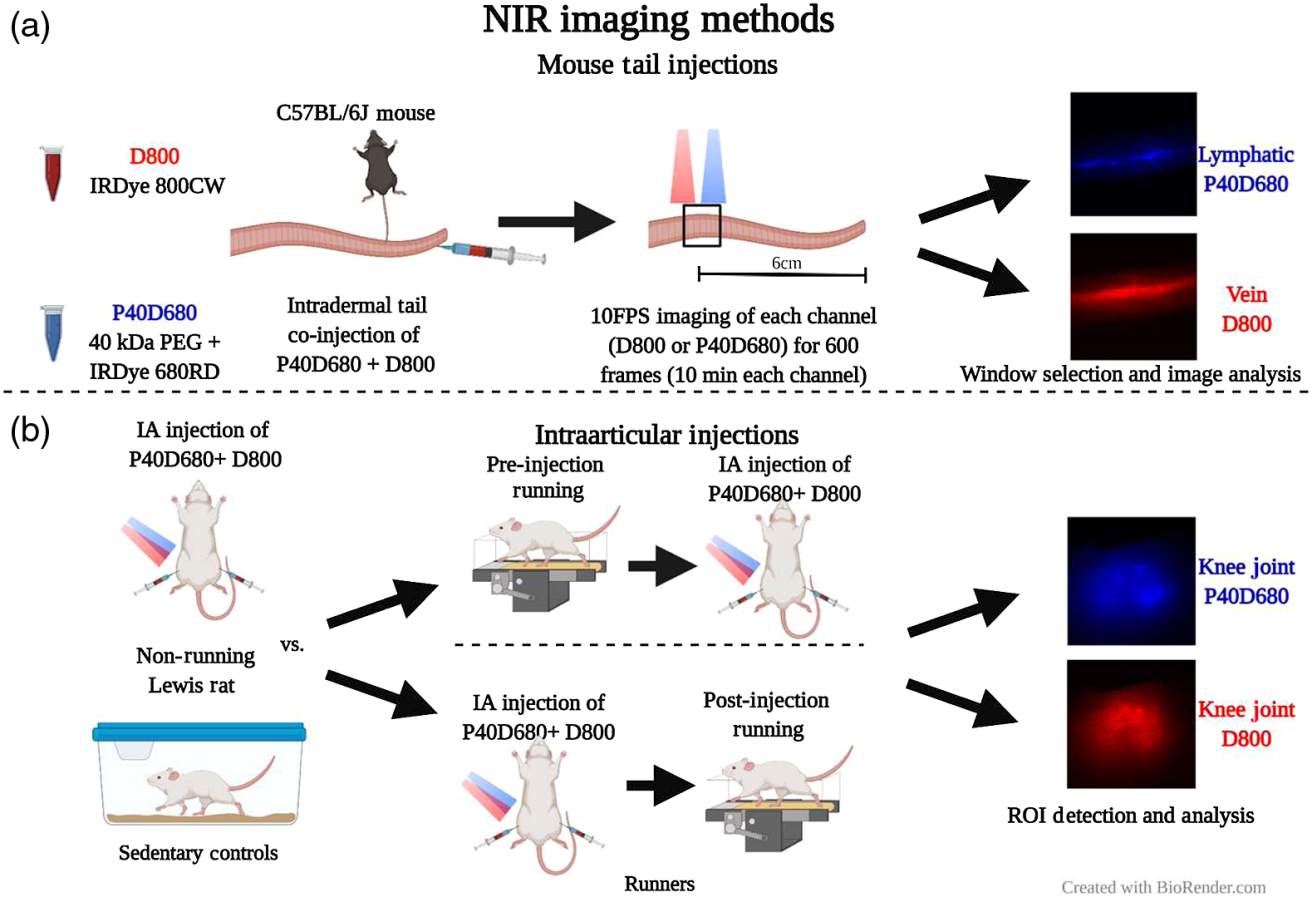
*In vivo* NIR imaging methods. (a) Mouse tail injection and imaging methods. (b) Intra-articular injections for assessing the effect of exercise on tracer clearance.

Animal care and experiments were conducted under the institutional guidelines of the Georgia Institute of Technology. Experimental procedures were approved by the Georgia Institute of Technology Institutional Animal Care and Use Committee (IACUC). To visualize the mouse tail lymphatics and blood vessels under bright-field, 20  μl of 1% (w/v) Evans blue solution was injected into the tip of the tail of an anesthetized mouse. Evans blue binds to interstitial proteins and was mainly taken up by lymphatics when injected intradermally. Posteuthanasia, the skin was removed at the base of the tail to reveal the underlying vasculature. Images of the vasculature were taken using a standard color camera to provide a comparison with NIR images. For NIR imaging through the skin, isoflurane was used to anesthetize C57Bl/6J mice, and the animal was placed in a recumbent position (on its side). A mixture containing 2.5  μl of the D800 and 2.5  μl of P40D680 was loaded into 1 ml insulin syringes (Becton Dickinson) and injected intradermally at the tip of mouse tails (two males and two females). After injection, the base and the end of the tail were taped to minimize motion artifact. D800 and P40D680 signals were imaged in 1-min, alternating increments [500 frames, Fig. 1(a)]. Two mice were initially imaged with the appropriate filter sets for P40D680 followed by D800, and two other mice were imaged with filter sets in the opposite order. Fluorescence images for both channels were acquired with 50 ms camera exposure time and 10 frames per second.

### 2.6 Intra-Articular Injections for Clearance

Animal care and experiments were conducted per the institutional guidelines of the Atlanta Veteran Affairs Medical Center (VAMC). Experimental procedures were approved by the Atlanta VAMC IACUC. Male Lewis rats weighing 350 to 400 g were treadmill trained 2 weeks prior to knee clearance studies. Training regime consisted of the following: on day 1, rats were acclimated to the treadmill for 30 min without running; on day 2, treadmill speed was set to 5  m/min for 5 min and 0  m/min for 25 min; each day thereafter, the time spent running was increased by 5 min increments until rats could run continuously for 30 min on consecutive days. Rats that failed to meet the targeted exercise regime were not used. Trained rats were randomly selected for control group (n=8) or exercise group (n=8). Three sets of studies were conducted: study (1): rats in the control group and rats in the exercise group were injected with D800 and P40D680 NIR tracers (no running) into the left and right knees; study (2): rats in the exercise group ran on the treadmill for 30 min at speed of 5  m/min before NIR tracers were injected into the left and right knees (pre-injection running), whereas rats in the control group did not receive any exercise that day and were injected with NIR tracers; study (3): rats ran on treadmill for 30 min immediately after injection of NIR tracers into the left and right knees (postinjection running), whereas rats in the control group did not receive any exercise that day and were injected with NIR tracers.

The day before initiation of the studies, rats were anesthetized, their hair from the knees and lower abdomen were removed, and background images of their knees were taken. NIR-tracers were injected in both knees and imaged at set time intervals (∼0, 1, 2, 3, 5, 7 12, and 24 h).

### 2.7 Image Processing and Analysis

Images captured using our custom NIR imaging system were saved as 16-bit, 512×512  pixel TIF files. Tracer’s intensity in the image was quantified using a customized MATLAB (MathWorks) script. The averaged fluorescence intensity within the region of interest (ROI) (5% highest intensity pixels) was calculated for each image and time point. This ROI corresponded to the knee joint space [Fig. 1(b)]. Data points were fitted to a monoexponential function $f(t)=y0+Ae^{-kt}$, where y0 is the offset, t is the time in hours, A is the normalized maximum fluorescence intensity, and k is the time constant. τ (tau) was determined as the inverse of k. To compare each intervention’s short-term effects, the mean change in ROI intensity over the first hour was calculated and subtracted from the mean value of the control rats. To determine each intervention’s overall effects, the time constant for each exercised rat was normalized to the control group.

For mouse tail injections studies, the filter was changed manually every 1 min. To remove imaging artifacts from the manual changing of the filter, 10 s (100 frames) during the filter switch were removed from the analysis. Fiji software was used to crop, register, and quantify ROIs.^27^ An ROI was drawn on the blood and lymphatic vessels to quantify the signals of D800 and P40D680, respectively. Furthermore, to quantify lymphatic uptake and transport of both tracers in the first (early, 0 to 10 min) and second (late, 10 to 20 min) half of imaging, one ROI was drawn over one of the two lymphatic vessels and lymphatic contractility metrics (frequency, packet transport, and packet integral) were assessed using a custom MATLAB code as described previously.^25^ Frequency was calculated as the number of contractions per minute, packet transport was calculated as the average normalized “packets” of fluid transported (a measure indicative of stroke volume), and the packet integral was the sum of the total packets over the imaging window (a measure indicative of volume flow rate due to contraction assuming no back flow).

### 2.8 Data Presentation and Statistics

For mouse tail studies, all venous and lymphatic data were separated and concatenated for analysis. A one-way ANOVA with Tukey’s multiple comparison test was used to compare all groups. All contractility and clearance data are presented as mean±SEM. A Brown–Forsythe test was used to quantify if variances were significantly different. A student’s t-test with a Welch’s correction was used to compare venous and lymphatic area under the curve (AUC) and tau in control rats. A one-way ANOVA with Dunnett’s multiple comparison test was used to calculate statistical significance for exercise group compared with control group.

## 3 Results

### 3.1 Optimization and Characterization of NIR Tracers Using an *In Vitro* Tissue Phantom

**Fig. 2:**
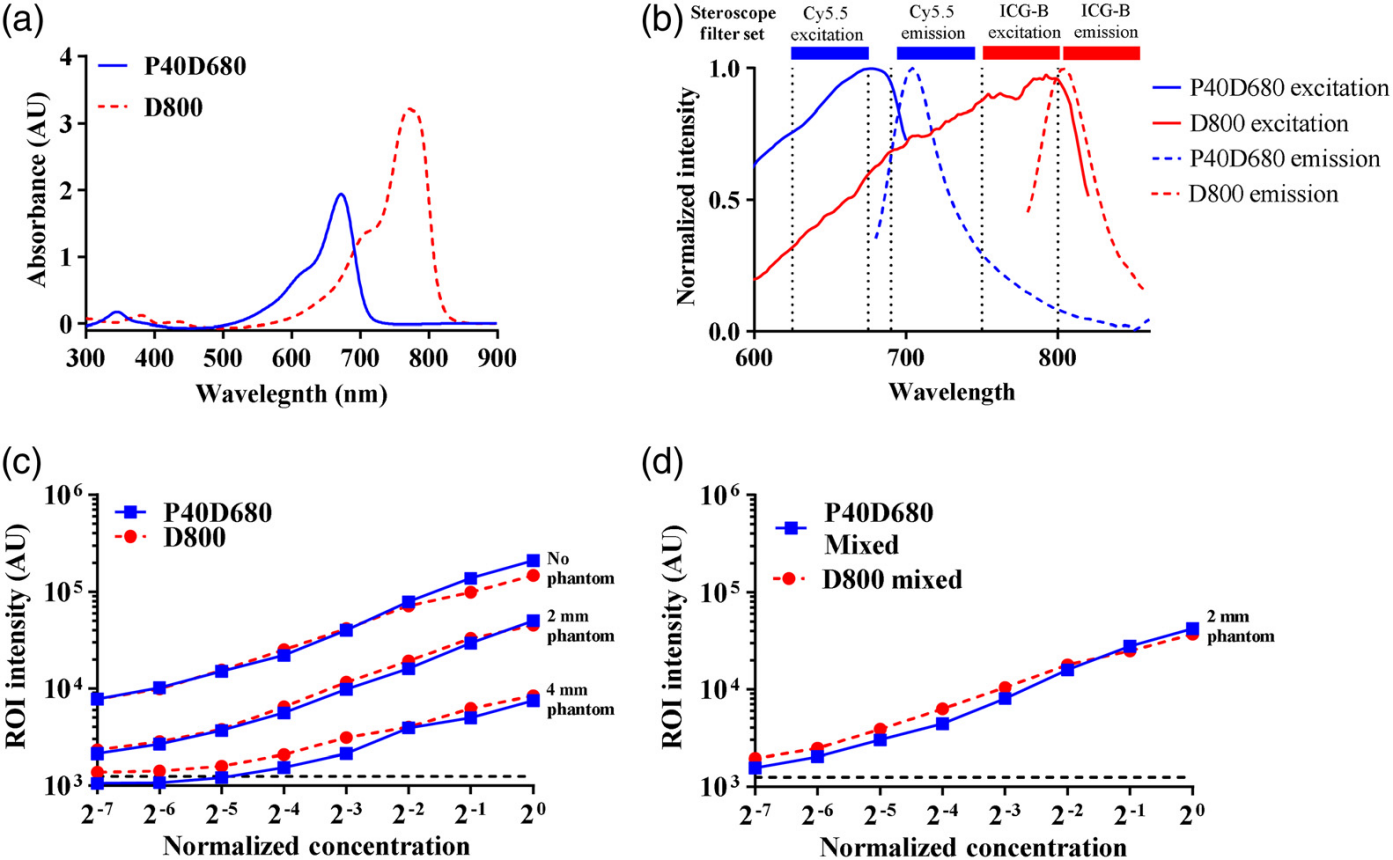
Sensitivity analysis of NIR dyes with tissue phantoms. (a) Absorbance spectra show the absorption profile for each tracer. (b) Solid and dashed lines show the excitation/emission spectra for 800CW carboxylate (D800) and IRDye 680RD conjugated 40 kDa PEG (P40D680), respectively. Our NIR stereoscope filter cube setup is represented by the bars above the graph, showing that our optical configuration is designed to read each tracer’s signal. (c) Tissue phantoms were used determine the effect of tissue depth on detection of D800 and P40D680. (d) D800 was serially diluted using a stock solution of P40D680 and vice versa. There was no change in overall intensity and sensitivity for each tracer due to mixing.

Absorbance spectra of D800 and P40D680 displayed an absorption maximum of 765 and 672 nm, respectively [Fig. 2(a)]. Emission and excitation spectra for these tracers also show each tracer’s expected maxima referenced to the full-width half maximum of the filter sets on the imaging system [Fig. 2(b)]. To determine the limits of detection in our imaging system as a function of concentration and tissue depth, we used tissue phantoms. Individual tracers were imaged in 1.5-ml centrifuge tubes with and without 2- and 4-mm phantoms. Increasing the tissue phantom thickness decreased the fluorescent intensity, though even at 4 mm, the dyes could be detected at a concentration of 3% of the injection concentration [Fig. 2(c)]. At a thickness of 2 mm, which is within the depth of most superficial lymphatics in the mouse tail, this detection limit was <1% of the injection site’s intensity. Notably, mixing the tracers did not affect the sensitivity to detect either fluorophore when contained in the background of the other via mixing [Fig. 2(d)].

### 3.2 NIR Tracers of Different Size Exit through Spatially Distinct Clearance Pathways

**Fig. 3:**
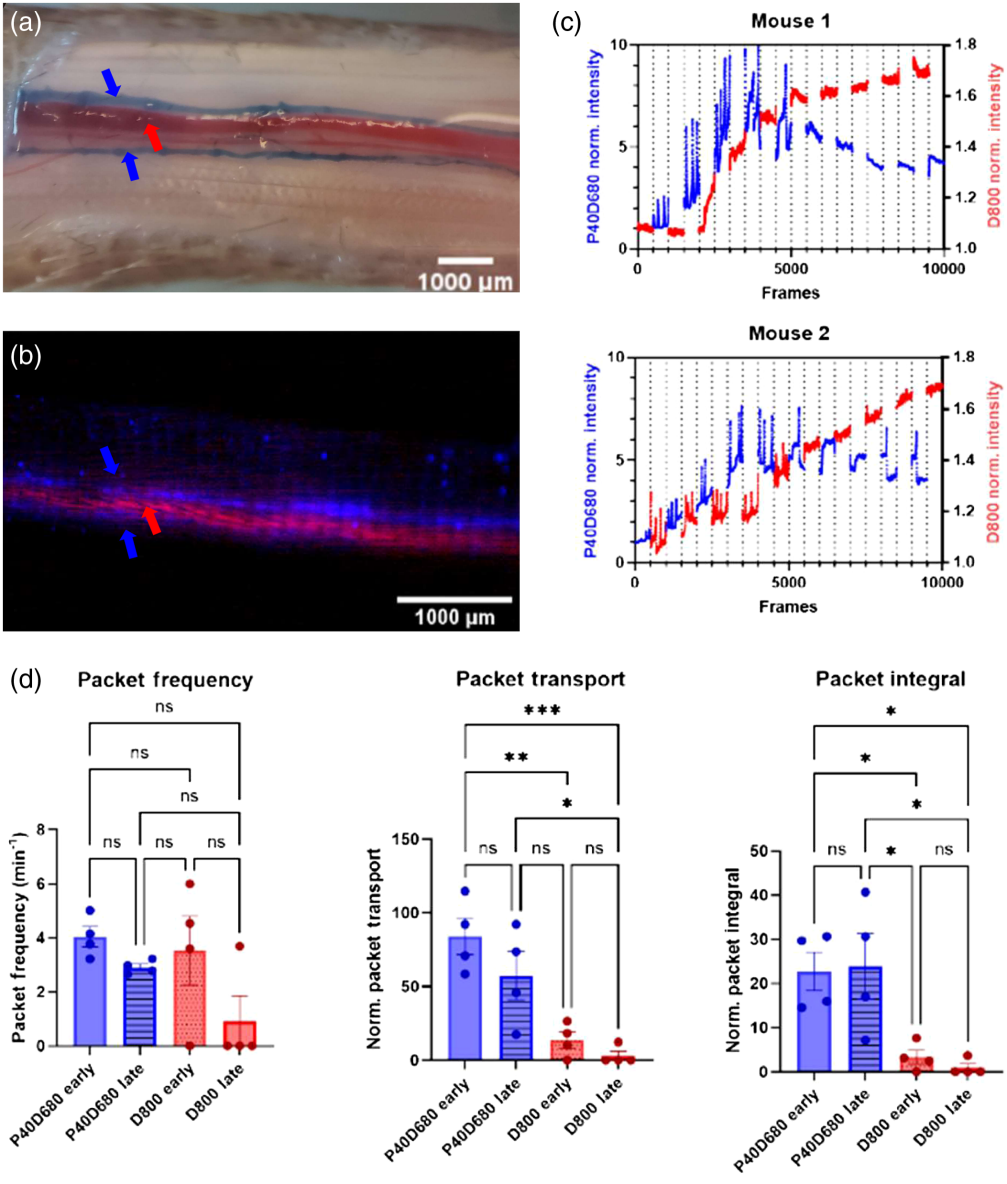
Coinjection of NIR tracers results in differential uptake of D800 and P40D680. (a) Evans blue dye injected into the tail of a mouse immediately before euthanasia. (a) shows the concentration of Evans blue dye was found in the lymphatics (blue arrows) that flanked the blood vessels (red arrows) (scale bar=1000  μm). (b) Overlayed images of D800 (red arrow) and P40D680 (blue arrows) show the dominant route of uptake of each NIR tracer in the vein and lymphatics 10 min post-injection in the mouse tail, with each frame for each tracer being captured ∼10  s apart from one another (scale bar=1000  μm). (c) Representative intensity curves for D800 and P40D680 in the venous and lymphatic circulation during imaging, respectively. Mouse 1 starts with the D800 filter and mouse 2 starts with the P40D680 filter set. (d) Quantification of lymphatic contractility metrics in the tail (n=4) shows strong functional contraction, as evidenced by packet transport and packet integral, of the PD40680 tracer but not the D800 tracer providing evidence that they are primarily in the lymphatic and venous circulation, respectively.

**Fig. 4:**
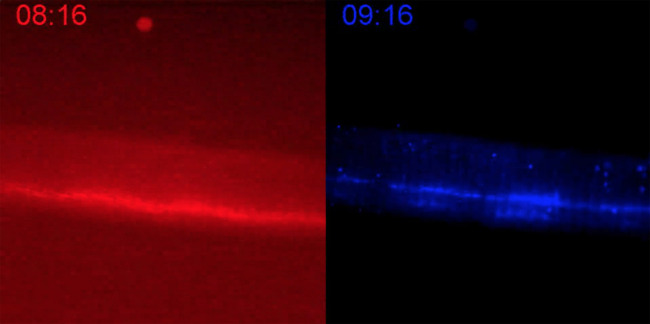
100FPS (10× speed) video of 5000 frames each of P40D680 (blue/right) and D800 (red/left) signal over 20 min of imaging for mouse 1. Propagation of fluorescent packets, due to lymphatic contraction, is evident in the P40D680 but not the D800 channel. Due to the interwoven nature of image acquisition, a timestamp is provided for each video to indicate when during the 20 min interval the images are acquired (Video 1, MP4, 5.6 MB [URL: https://doi.org/10.1117/1.JBO.26.12.126001.1]).

The mouse tail circulatory and lymphatic vasculature were visualized via intradermal injection of Evans blue and after removal of the skin [Fig. 3(a)]. The lymphatics took up Evans blue, which allowed clear visualization of the two lymphatic vessels running parallel to the tail vein and artery. To simultaneously quantify lymphatic and venous drainage, these tracers were co-injected at the end of the mouse tail and images for each tracer were captured upstream from the injection site. Figure 3(b) showed that D800 was primarily taken up into the blood circulation (red arrow), and P40D680 was primarily found in the lymphatics (blue arrows). The dyes were spatially distinct and reflected the expected anatomy, i.e., two lymphatic vessels flanking a major blood vessel in the mouse tail. Representative curves of D800 and of P40D680 signals are shown in Fig. 3(c). The fluorescence intensity of each tracer increased over time and reached near equilibrium by 10 min (5000 frames). The order in which the filter sets were used did not influence the general trends in the kinetics of drainage. Lymphatic contractions (as represented by P40D680 signal spike) were detectable within the first 10 min of imaging; however, contractions were less dynamic at later times in agreement with previously published observations in the rat tail.^16^ D800 signal steadily increased in the large vessel as the tracer accumulates in the circulation. When lymphatic contraction parameters were quantified for each respective tracer in the first and second half of each imaging session [Fig. 3(d)], P40D680 showed continuous contraction throughout the entire experiment (particular as evidenced by packet integral), whereas D800 shows some contractility immediately after injection (likely due to a similar concentration of dye in blood and lymph early on), and very little detectable contractility at later time points as the presence of the small tracer in the blood dominates the signal. Even at early time points, a direct comparison between the normalized packet integral of P40D680 compared with D800 [Fig. 3(d)] reveals that the larger tracer relies much more heavily on lymphatic transport than the smaller tracer for its tissue clearance. This is also shown in the video associated with Fig. 4 at 10× speed for mouse 1.

### 3.3 Co-Injection to Assess Differential Tracer Clearance in the Joint

**Fig. 5:**
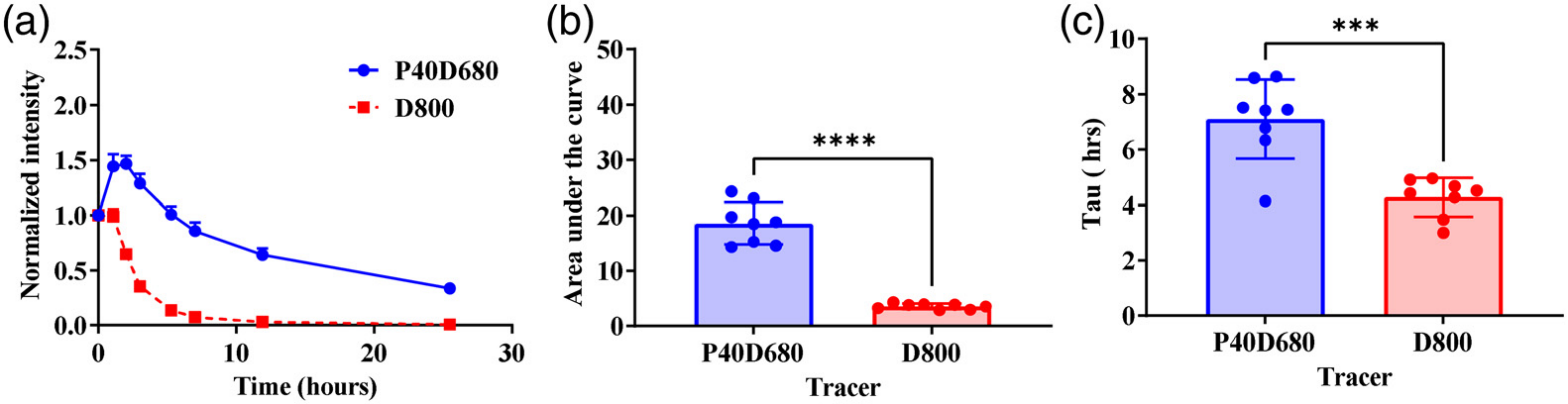
Co-injection of NIR tracers enables simultaneous detection of lymphatic and vascular mediated clearance from the joint space. (a) Clearance profiles for P40D680 and D800 show characteristic lymphatic and venous clearance from the rat joint space using NIR imaging, respectively (n=4 rats, both knees). (b) The areas under the curves show a significantly lower AUC for D800 compared with the P40D680 (**,p≤0.01). (c) First-order clearance constant tau was calculated for each tracer and is significantly higher for the lymphatic draining P40D680 compared with the venous draining D800.

The effect of exercise on intra-articular clearance has not been extensively studied,^28^^,^^29^ specifically to quantify the change in venous and lymphatic drainage in the same joint. Therefore, after confirming size-dependent uptake from the tail, we used multichromatic imaging to assess intra-articular drainage. Unlike intradermal injections, materials from the joint space clear much slower;^30^^–^^32^ therefore, venous and lymphatic clearance from this interstitial depot was expected to occur over 1 day. Simultaneously injected tracer clearance P40D680 and D800 profiles [Fig. 5(a)] exhibited an initial increase followed by monoexponential clearance kinetics consistent with previous reports.^24^ Specifically, the lymphatic tracer P40D680 showed a substantial increase in intensity after the injection, whereas this increase was less pronounced for the venous tracer D800. By 12 h postinjection, the intensity of the D800 was not detectable [Fig. 5(a)]. The normalized AUC was calculated to be 3.59±0.18 for D800 and 18.57±1.33 for P40D680 (p<0.0001), demonstrating slower clearance of the P40D680 from the joint space [Fig. 5(b)]. The time constant (Tau) for the clearance of D800 was 4.28±0.25  h (n=8), whereas the time constant for P40D680 was 7.11±0.51  h (n=8;p=0.0003) [Fig. 5(c)]. After 24 h, P40D680 tracer was still detectable within the joint [Fig. 5(a)], likely due to some P40D680 tracer remaining trapped in the solid matrix of the interstitium.

### 3.4 Multichromatic Imaging for Measuring Patterns in Joint Clearance

**Fig. 6:**
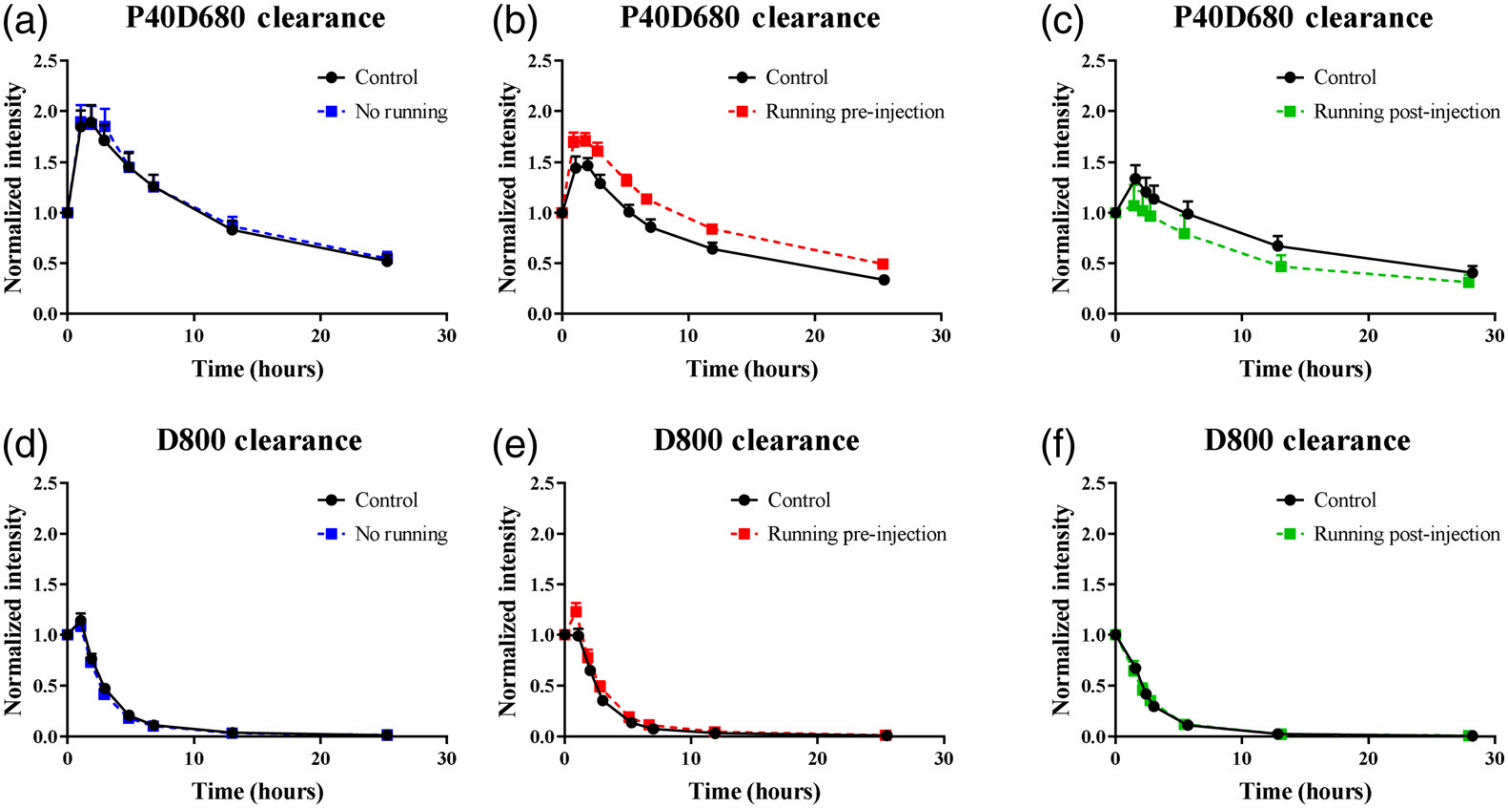
Clearance profiles for P40D680 and D800 with exercise. (a)–(c) Clearance profiles of the lymphatic specific tracer (P40D680) for no exercise, exercised pre-injection running, and exercised postinjection studies. (d)–(f) Clearance profiles for venous draining (D800) for no exercise, exercised pre-injection running, and exercised postinjection studies. No running, pre-injection running, and postinjection running experiments (n=8 for control group and n=8 for each experimental group).

**Fig. 7:**
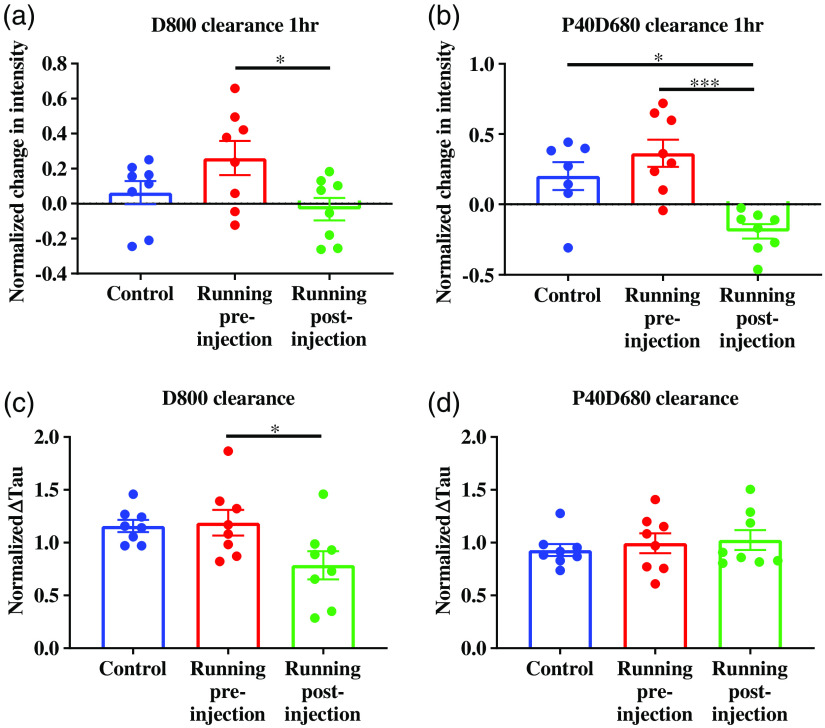
Effect of exercise on normalized change in intensity and normalized Tau. (a), (b) The normalized intensity at 1 h was calculated to assess the transient effect of exercise on tracer dispersion within the joint. For each study, each exercised rat was normalized to the mean of non-running the controls. (* in (a), p=0.04, * in (b), p=0.01, and *** in (b), p=0.0003.) (c), (d) Time constant (tau) for each condition was normalized to the controls for each day. (* in (c), p=0.04). (n=8 rats for control and experimental groups.)

To demonstrate the utility and sensitivity of multichromatic imaging to evaluate differential changes in clearance mechanisms for venous and lymphatic drainage, trained rats were exercised (5  m/min for 30 min) either pre- or postinjection and were compared with trained, nonexercised rats conducted within the same study. Trained rats were randomly assigned to control group or test group (nonexercised, exercised pre-injection, and exercised postinjection). In rats that received no exercised intervention whatsoever, both lymphatic and venous clearance profiles were similar among the two groups [Figs. 6(a) and 6(d)]. However, the clearance curves for the exercised animals appeared to be qualitatively different from the nonexercised animals [Figs. 6(b), 6(c), 6(e), and 6(f)]. To quantitatively evaluate these differences, the normalized changes in fluorescence intensity were calculated [Figs. 7(a) and 7(b)], as well as the time constant [Figs. 7(c) and 7(d)]. For D800 clearance, the exercised pre-injection rats (0.26±0.10) exhibited a significant increased (p=0.04) change in fluorescence intensity (at 1 h) compared with exercised postinjection rats (−0.03±0.06); however, neither groups were significantly different from nonexercised group (0.06±0.06) [Fig. 7(a)]. The normalized tau for D800 clearance for exercised postinjection group was 0.79±0.13 which was a significant reduction (p=0.04) compared with exercised pre-injection group (1.188±0.12) [Fig. 7(c)]; however, neither was significantly different from nonexercised group (1.16±0.05).

When assessing lymphatic clearance, we observed a significant reduction (p=0.0003 and p=0.01) in the initial change in fluorescent intensity (at 1 h) in exercised postinjection (−0.19±0.05) compared with exercised pre-injection (0.36±0.1) and nonexercised controls (0.20±0.1) [Fig. 7(b)]. There was no significant change in tau for P40D680 tracers among the three groups [Fig. 7(d)]. These data suggest the transient effect of exercise may be lost over the much longer timescale for which lymphatic clearance occurs.

## 4 Discussion

In this paper, we applied multichromatic NIR imaging to assess interstitial clearance mechanisms from multiple tissue beds. Clearance pathways and rates are essential in tissue homeostasis and dictate how biomolecules interact with their intended targets. Clearance to lymphatics or to venous circulation is understudied *in vivo*. Using tissue phantoms, we established the exposure time and tissue depth limitations required for *in vivo* imaging. We demonstrated that the two tracers, D800 and P40D680, do not affect each other’s fluorescence properties and thus can be used to independently ascertain differing clearance pathways. In the mouse tail, a tissue drainage bed with well-defined physiology, lymphatics, and venous circulation may be visualized *in vivo*, and their function may be measured using P40D680 and D800, respectively. Further, we demonstrated the capacity to quantitatively image routes of clearance from the knee joint space. We utilized exercise-based intervention to alter the clearance of D800 and P40D680 tracers.

Our study demonstrated the size dependence of interstitial molecules via venous and lymphatic pathways via simultaneous imaging. In our mouse tail study, the D800 intensity in the blood ROI is initially low. The D800 signal increases in the tail vein over time, likely due to a renal clearance not surpassing the intradermal depot clearance over this total imaging window.^33^ Thus, D800 dye intensity in the blood circulation continuously increases as the concentration delivered into the blood stream as time increases. The P40D680 tracer signal intensity traces exhibited the characteristic phasic contractions attributed to lymphatic pumping.^17^^,^^25^ Therefore, our mouse tail experiment validated our two tracers’ size-based partitioning to distinct routes of clearance.

The joint space is a unique interstitial space comprised of synovial fluid—hyaluronic acid, lubricin, and filtered serum—that hydrates the joint tissues and buffers the outflow of materials from the joint space.^34^^,^^35^ A solute that leaves the joint space must diffuse through the synovial fluid then into the synovial membrane. The synovial membrane is a specialized tissue that retains the synovial fluid while also housing the venous and lymphatic fluid exchange machinery to clear solute from the joint.^25^^,^^36^ Smaller materials can more easily diffuse through the synovial fluid matrix and thus exit the joint space faster.^37^ Larger molecules may entangle in the synovial fluid matrix and therefore may have longer residence time within the joint space.^38^ Using multichromatic imaging with differing sized tracers enables simultaneous quantification of venous and lymphatic clearance kinetics in the joint rather than separately as conducted in previous studies^20^ and furthers the ability to determine the relationship between lymphatic and venous uptake *in vivo*.

Exercise has been shown in previous studies to increase interstitial,^39^ venous,^40^ and lymphatic^41^ flow to the muscle. In this study, we used exercise as an intervention to demonstrate the sensitivity of multichromatic NIR imaging to measure changes to venous and lymphatic clearance in the knee joint. In the joint space, exercise and joint loading have increased intra-articular pressure and cartilage fluid flux. In this study, injection of tracers following exercise transiently increased lymphatic outflow from the joint; however, this exercise regime did not have a measurable effect on venous clearance. Interestingly, exercised pre-injection led to delayed clearance of both D800 and P40D680, as exhibited by the presence of a larger peak intensity from the joint than their respective controls, which could be a consequence of altered hydrodynamic forces or delayed dye dispersion. It is well known that various cytokines and blood pressure can alter lymphatic contractility^42^^,^^43^ and that these are likely altered during exercise,^44^^,^^45^ which could be a potential mechanism underlying the transient effect of exercise observed on lymphatic clearance. In work that is currently ongoing, our group has shown that exercise leads to the decreased levels of certain vasoactive cytokines within the joint (unpublished). Given that the routes of clearance for these vasoactive cytokines are through these two vascular pathways, exercise could have effects on lymphatic and venous contractility and tone via changes in joint cytokines, which is an important area of future study.

Our current setup limited our temporal sampling frequency in both mouse and rat *in vivo* studies. For exercise experiments, sampling was limited by the time required to anesthetize the animal for NIR imaging and the transient effect of exercise could be not captured at later time points beyond 1 h. An ideal setup would be a wearable sensor that would go around the knee, which would allow real-time imaging without requiring anesthesia. Similarly, in capturing the routes of clearance in the mouse tail, only one channel could be used at a given time. Two cameras and light paths, or a computerized filter wheel, would enable simultaneous assessment of two tracers with higher temporal frequency to better quantify the relationship between vascular and lymphatic uptake *in vivo*. In addition, the tracer sizes were designed to evaluate the particulate transport within fluid; however, there are also cell-mediated mechanisms by which transport occurs *in vivo,* which could be imaged using these multichromatic approaches.

We conclude that multichromatic NIR imaging is capable of simultaneous imaging of lymphatic and venous-mediated fluid clearance with great sensitivity and can be used to measure transient changes in clearance rates and pathways. The fluorophores and materials could be refined to provide more colors and construct sizes in the NIR range for 3 or 4 color imaging modalities. The NIR-II imaging window could be used to visualize deeper structures *in vivo.* This methodology can be applied in future studies that assess the effects of diseases or surgical interventions on interstitial solute transport and tissue fluid homeostasis.

## Code, Data, and Materials Availability

The raw data for this study were generated at Georgia Tech and Emory University. Data or materials supporting this study’s findings are available from the corresponding authors BD and NW.
